# Crashworthiness Performance of Bamboo-Inspired 3D-Printed Tubes: Effects of Infill Pattern, Infill Ratio, Wall Thickness, and Inner Diameter

**DOI:** 10.3390/biomimetics10100702

**Published:** 2025-10-17

**Authors:** Emre İsa Albak

**Affiliations:** Automotive Engineering Department, Engineering Faculty, Bursa Uludağ University, Bursa 16059, Türkiye; emrealbak@uludag.edu.tr

**Keywords:** bamboo-inspired design, bio-inspired structures, crashworthiness, 3D printing, infill pattern, infill ratio, quasi-static compression test

## Abstract

This study investigates the impact absorption performance of bamboo-inspired 3D-printed circular tubes in terms of infill type (grid, gyroid, honeycomb, Archimedean chords), infill ratio (10%, 20%, 30%, and 40%), wall thickness (0.8, 1.2, 1.6, and 2.0 mm), and inner diameter parameters. The structures designed using Taguchi L16 orthogonal design are printed with PLA material using FDM technology and evaluated by quasi-static compression tests. Peak crushing force (PCF), energy absorption (EA), and specific energy absorption (SEA) criteria are used to analyse crashworthiness performance. The experimental results showed that EA improves as the infill rate increases, but the gain decreases as the infill rate approaches 40% (SEA at 30% is better than that at 40%). By visualising the PCF and EA data relative to the utopia point (the lowest PCF and highest EA), GRID_T16F30D24, HCOMB_T08F30D22, and GRID_T12F20D22 are found to be the best-performing tubes. The grid and honeycomb infill types showed superiority at 20–30% infill rates and similar wall thicknesses. The Archimedean chords type performed poorly due to its tendency to fracture.

## 1. Introduction

In recent years, the demand for lightweight and impact-absorbing structural elements has increased significantly in transportation systems in line with the requirements to increase passenger safety and improve fuel efficiency. In this respect, thin-walled energy-absorbing structures have come to the forefront, and their crashworthiness performance depends on many parameters such as geometry, material selection, and internal structural configurations. Additive manufacturing (AM), particularly fused filament fabrication (FFF), provides an innovative approach to achieving the desired crashworthiness characteristics with various material alternatives and geometric configurations.

Different materials such as ABS [1], ASA [2], PET [3], PETG [4], and PLA [5] are used by researchers to improve impact damping and mechanical behaviour with additive manufacturing. Polylactic acid (PLA), a biodegradable thermoplastic widely used in additive manufacturing, excels in impact applications due to its processability, dimensional stability, and acceptable energy absorption capacity [6,7,8]. Alaziz et al. [9] investigated the crashworthiness characteristics of circular, lightweight, impact-absorbing advanced polylactic acid (PLA+) structures inspired by the natural morphology of the horsetail plant. Tunay et al. [10] experimentally investigated the quasi-static crashworthiness performance of sandwich panels with four different 3D-printed core geometries: honeycomb, re-entrant, double-arrowhead, and missing rib-cut. These core geometries were produced using the FDM method with PLA and ABS materials and bonded to CFRP surface layers. As a result of their study, the missing rib-cut geometry achieved the highest EA, reaching values of 85.25 (PLA) and 52.85 J (ABS), while in all geometries, PLA-based cores outperformed ABS-based cores in EA. Isaac et al. [11] experimentally investigated the crashworthiness characteristics of impact absorbers with a honeycomb structure of different infill ratios (20%, 40%, and 60%) using PLA, ABS, ASA, PETG, and NCC materials under quasi-static conditions. According to the experimental results, PETG and PLA honeycomb structures exhibited similar values in terms of both EA and SEA, whereas these two structures had higher EA and SEA values compared to honeycomb structures made of ABS, ASA, and NCC materials.

Different geometric approaches are focused on improving the crashworthiness performance, especially in terms of energy absorption, of impact-absorbing structures produced by additive manufacturing. Infill type and infill ratio are among the important factors examined [12,13,14,15]. Bandinelli et al. [16] investigated four different infill lattice structures (gyroid, Cross3D, Octet, and cubic) manufactured via fused deposition modelling (FDM), through compression tests by varying both the weight density (200 g/L and 300 g/L, comparable to low-density aluminium foams) and the strain rate (0.01 s^−1^ and 1 s^−1^). Aloyaydi et al. [17] experimentally investigated the crashworthiness characteristics of circular impact absorber structures filled with PLA material using triangle, grid, quarter cubic, and tri-hexagon infill patterns under low-velocity impact test conditions, and they found that the triangle pattern achieved the highest peak force of 1190.5 N and penetrating energy of 7.50 J due to its effective raster and layer bonding, greater contact points per unit area, and increased sheared layers, resulting in improved mechanical response. Awd Allah et al. [18] investigated the quasi-static axial crashworthiness behaviour of 3D-printed PLA square tubes by varying three main printing parameters: filler pattern (gyroid, honeycomb, Schwarz P, Schwarz D), filler density (5–30%), and layer height (0.15–0.30 mm). Their analysis revealed that the combination of a honeycomb pattern, 30% infill density, and a 0.20 mm layer height exhibited the best crashworthiness in all criteria. Awd Allah et al. [19] experimentally investigated the crashworthiness characteristics of circular structures under quasi-static conditions using three infill configurations, circular, square, and triangular, with infill densities of 30%, 50%, and 70%. Their study revealed that infill type and density have significant effects on impact performance.

Although previous studies have extensively investigated the individual and combined effects of infill pattern and infill ratio on the crashworthiness of 3D-printed structures [12,13,14,15,16,17,18,19], a systematic and simultaneous exploration of the interplay between infill pattern, infill ratio, wall thickness, and inner diameter remains limited. In particular, the role of wall thickness and inner diameter as independent geometric variables—distinct from infill density—has not been thoroughly examined within a unified experimental framework. This study addresses this gap by adopting a bamboo-inspired design philosophy not merely as a bio-inspired motif but as a functional rationale for integrating these four parameters into a cohesive structural system. The hierarchical, graded, and porous internal architecture of bamboo culms [20,21,22,23] provides a biomimetic blueprint for optimising energy absorption through controlled wall thickness, internal void distribution (inner diameter), and cellular infill geometry. In this analogy, the stiff outer wall mimics the dense sclerenchyma layer of bamboo, while the internal infill patterns (grid, gyroid, honeycomb) emulate the function of the fibrous vascular bundles and porous parenchyma cells that contribute to its high specific energy absorption. Thus, a four-level experimental design with a total of four design variables is created, and investigations are conducted on 16 different specimens using the Taguchi L16 orthogonal array design method. The crashworthiness performance of the specimens is determined through experimental studies conducted under quasi-static axial loading conditions. Through this comprehensive approach, the individual and interactive effects of not only the infill structure but also the key geometrical parameters of the impact-absorbing structures on energy absorption performance are thoroughly investigated.

## 2. Experimental Procedure

### 2.1. Bamboo-Inspired Design

In studies aimed at improving the crashworthiness performance of impact-absorbing structures, researchers frequently prefer nature-inspired designs. The durable structure of bamboo, in particular, is among the most frequently cited structures for improving crashworthiness performance [20,21,22]. In this study, circular-section energy-absorbing structures are designed using a 3D printer, inspired by the cross-sectional structure of the bamboo plant. This design approach, which mimics the porous and graded internal structure of natural bamboo, aims to increase energy absorption capacity during a crash. The designed samples have a circular geometry, and all samples have a fixed outer diameter of 40 mm and a height of 60 mm.

**Figure 1:**
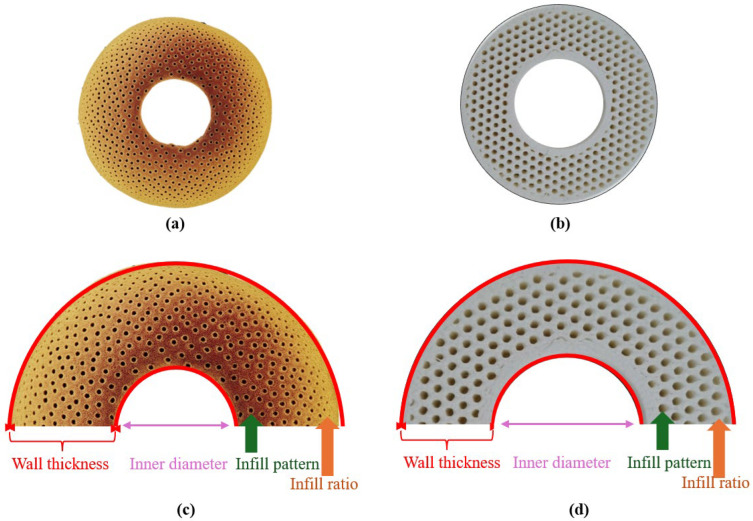
Bamboo-inspired design and visualisation of variable parameters for circular impact-absorbing structure inspired by bamboo. (**a**) Natural bamboo cross-section [23]. (**b**) Cross-section example of 3D-printed tube. (**c**) Bio-inspiration from bamboo culms. (**d**) Explanation of design parameters (wall thickness, internal diameter, infill pattern, and fill ratio).

Geometrical variability is achieved through four basic parameters (Figure 1).

*Wall thickness*: Wall thickness is the radial thickness of the outer shell, which plays a significant role in the structure’s rigidity and initial impact force. It is examined at four levels: 0.80 mm, 1.20 mm, 1.60 mm, and 2.00 mm.

*Inner diameter*: Inner diameter refers to the void at the centre of the structure. It influences the void ratio and mass distribution, thereby altering deformation patterns. It is applied at four different levels: 18 mm, 20 mm, 22 mm, and 24 mm.

*Infill pattern*: Infill pattern defines the geometry of the internal cellular structure and imparts properties similar to the porous structure of bamboo. Four different patterns are used in this study: grid, gyroid, honeycomb, and Archimedean chords.

*Infill ratio*: The infill ratio is the parameter that determines the density of the internal structure. It directly affects specific energy absorption (SEA) and the maximum impact force (PCF). The level range is as follows: 10%, 20%, 30%, and 40%.

Since each parameter has four levels, 4^4^ = 256 combinations could be obtained with the classic full factorial design. However, to reduce the experimental load, the Taguchi L16 orthogonal array design is preferred. This allowed for a statistical evaluation of the effects of all parameters with only 16 experimental conditions. The parameter levels are presented in Table 1, and the sample combinations created according to the L16 design are presented in Table 2.

**Figure 2:**
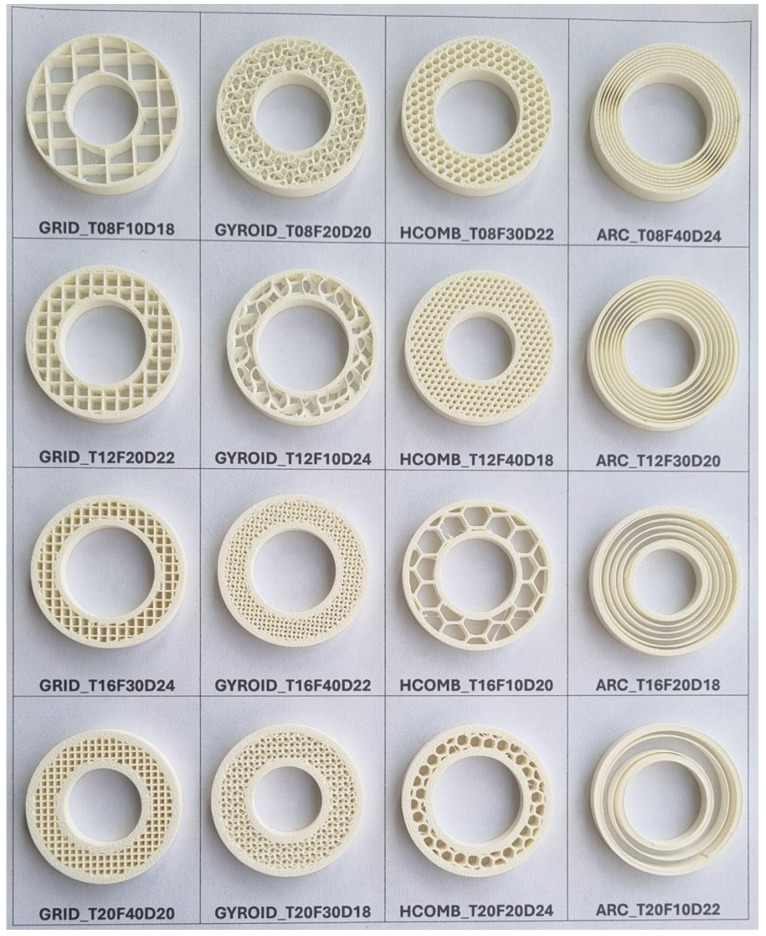
Images of 5 mm high specimen.

Additionally, 5 mm high samples are produced to visually identify each configuration and are presented in Figure 2. These samples facilitate both monitoring print quality and matching parameters with model labels. For example, a sample labelled GYROID_T08F20D20 indicates a gyroid infill structure, 0.80 mm wall thickness, 20% infill, and 20 mm internal diameter.

### 2.2. Three-Dimensional Printing

All samples are produced using fused deposition modelling (FDM) technology with the Bambu Lab X1E, a 3D printer designed for high-precision and multi-material production. The geometric properties of the circular structures’ wall thickness and inner diameter are modelled using a CAD-based drawing program. The infill patterns and infill ratios to be used during production are defined through Bambu Studio 2.2.2.56, Bambu Lab’s own production software. This software allows for the precise adjustment of the infill structures and controlled implementation of the printing protocol. A standard nozzle tip with a diameter of 0.4 mm is used in the production processes, and all samples are printed with the same production parameters, ensuring the reliability of inter-parameter comparisons. The basic parameters used in the 3D manufacturing process are given in Table 3. All specimens used in the experimental study are manufactured with a polylactic acid (PLA)-based filament material supplied by BASF.

### 2.3. Crashworthiness Criteria

Crashworthiness refers to the ability of vehicle structures to provide occupant safety and absorb energy during impact. In this context, various criteria have been developed in the literature, allowing for the quantitative evaluation of the impact performance of impact absorbers [24,25,26,27,28]. In this study, four main criteria commonly used in crashworthiness performance evaluations are examined.

*Peak Crushing Force (PCF):* Peak crushing force refers to the peak force value of the structure during impact. This value reflects the stiffness characteristic of the structure and its capacity to absorb sudden impacts.

*Energy Absorption (EA):* Energy absorption defines the total amount of energy absorbed by the structure during the deformation process. High EA values indicate effective energy absorption behaviour.

*Specific Energy Absorption (SEA):* Specific energy absorption is the ratio of absorbed energy to unit mass. It is an important criterion for lightweight and high-performance structures and provides an evaluation in terms of weight–efficiency balance.

Through these criteria, the impact strengths of the structural configurations examined are objectively analysed and contribute to the determination of optimum design parameters.

### 2.4. Quasi-Static Experiment

A universal testing machine with a capacity of 200 kN is used to conduct quasi-static axial compression tests on the 3D-printed specimens. These tests are designed to evaluate the mechanical behaviour of the specimens under controlled compressive loading conditions. During each experiment, the specimens are subjected to a total compression of 42 mm, applied at a constant deformation rate of 5 mm/min [29,30] to ensure quasi-static conditions. Data acquisition is performed at a sampling rate of 100 Hz, capturing detailed force and displacement measurements throughout the test. The collected data is subsequently processed using a filtering technique to remove noise and ensure accuracy.

The quasi-static testing approach is selected for this comparative study because it enables the clear identification of deformation mechanisms and the reliable measurement of crashworthiness parameters without strain rate complications. While actual crash events involve dynamic loading, quasi-static conditions provide a validated methodology for the initial screening and ranking of energy absorber designs, where the relative performance trends typically correlate well with dynamic behaviour. This approach offers a practical foundation for identifying optimal configurations before proceeding to more complex dynamic validation in future work.

## 3. Results and Discussions

### 3.1. Deformation Modes and Impact Force–Crushing Displacement Curves

**Figure 3:**
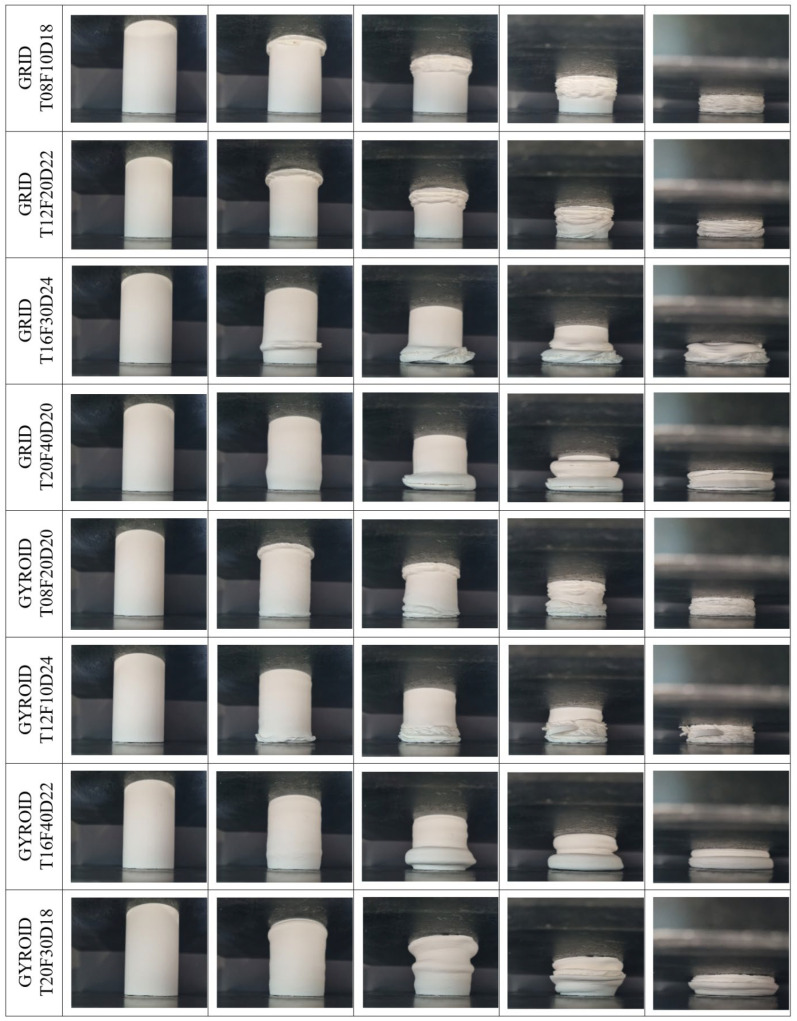
Compression test images of structures with grid and gyroid infill patterns.

**Figure 4:**
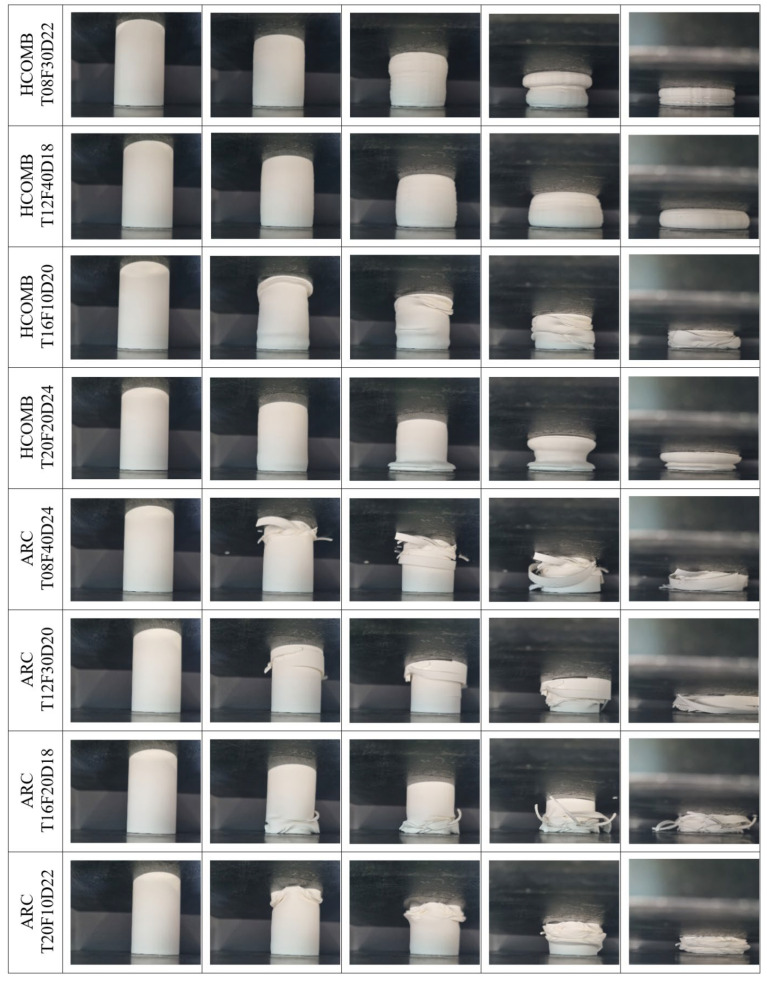
Compression test images of structures with honeycomb and Archimedean chords infill patterns.

**Figure 5:**
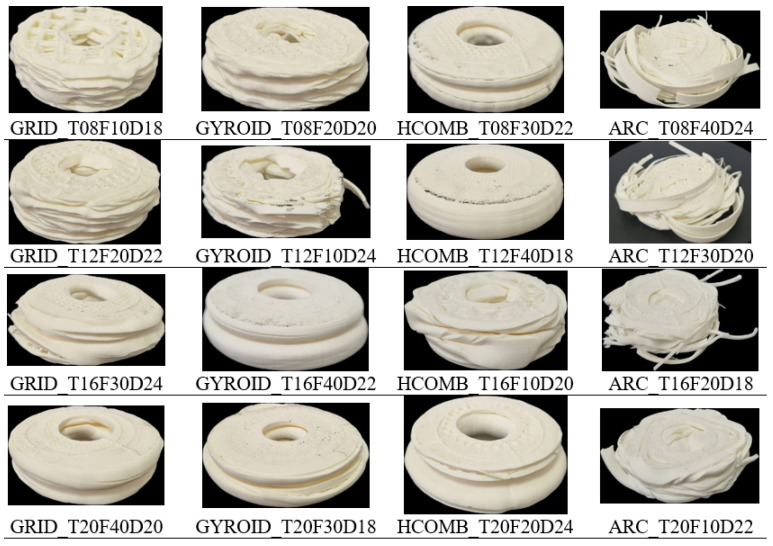
The deformed form of all models.

**Figure 6:**
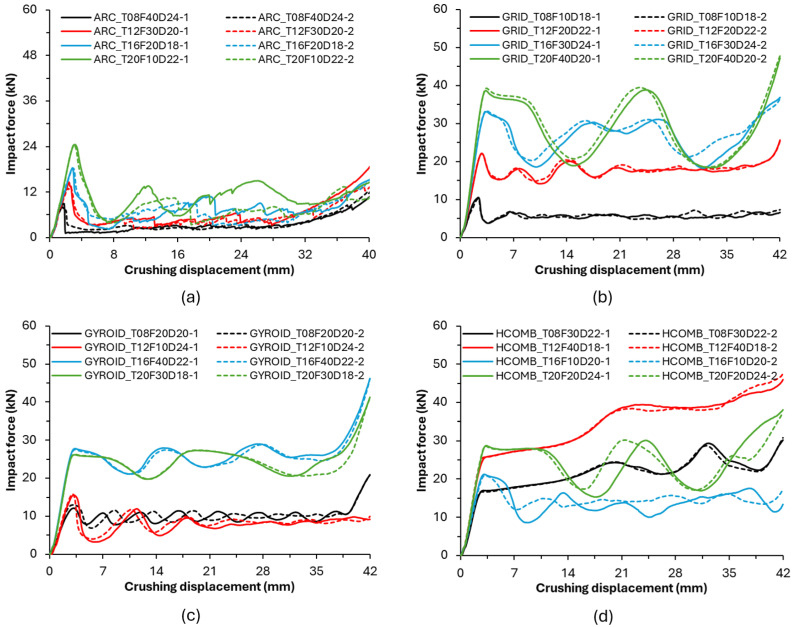
Impact force–crushing displacement curves of bamboo-inspired circular tubes. (**a**) Archimedean chords infill pattern. (**b**) Grid-type infill pattern. (**c**) Gyroid-type infill pattern. (**d**) Honeycomb-type infill pattern.

In this section, the results obtained in the compression experiments performed under quasi-static conditions with a constant speed of 5 mm/min of the parts created with the Taguchi L16 orthogonal array and produced in a 3D printer will be examined in detail. In this section, first, the deformation modes and force–displacement curves will be explained, and then the effects of the parameters will be analysed with Taguchi. The deformation modes of the bamboo-inspired circular structures during compression tests are given in Figure 3 and Figure 4. The deformed form of all models at the end of the compression tests is also given in Figure 5. The impact force–crushing displacement curves of the bamboo-inspired circular tubes are also given in Figure 6.

To evaluate the reproducibility of the experiments, each test configuration is repeated twice. Since the deformation modes observed in both repetitions are consistent with each other, the deformation patterns are presented representatively based on a single specimen. In contrast, the force–displacement curves for each test are plotted separately, allowing for a direct comparison of consistency in behaviour.

The results show that the force–displacement curves obtained for the grid, gyroid, and honeycomb infill structures are highly consistent in both repetitions, indicating that the mechanical response of these structures is highly repeatable. However, in the Archimedean chords infill type, noticeable differences are observed between repetitions, particularly in parameters such as peak force and crushing profile. The fundamental reason for this inconsistency may be attributed to the Archimedean chords-type pattern having weaker connection points in its internal architecture compared to other patterns. This structural characteristic prevents the elements from adequately supporting each other during deformation, leading to greater variation in mechanical performance.

**Figure 7:**
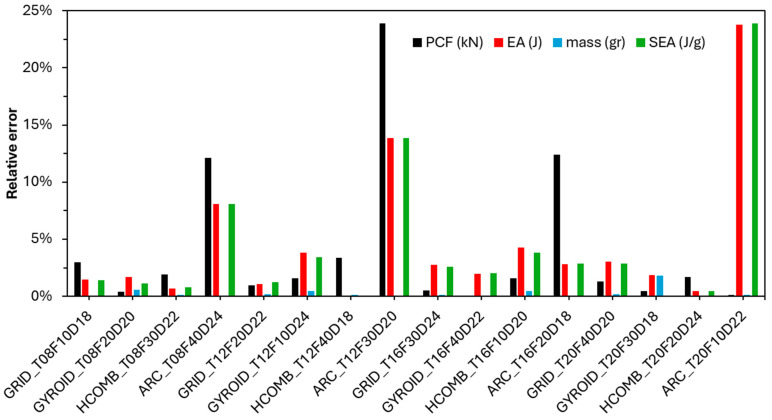
Relative error distribution of PCF, EA, mass, and SEA parameters.

The repeatability of the experimental results is also evaluated using the relative error graph presented in Figure 7. The graph shows that the relative errors for the peak force (PCF), energy absorption (EA), mass, and specific energy absorption (SEA) parameters are acceptable and low, except for the Archimedes pattern type. This supports the reliability of our experimental methodology and results.

When both crushing modes and force displacement curves are analysed, the worst infill pattern is seen to be “Archimedean chords”. In Archimedean chords-type patterns, at the beginning of crushing, the structures show fracture behaviour instead of plastic deformation. This is also seen in the impact force–crushing displacement curves in Figure 6. After the first peak force value, sudden decreases are observed. In other patterns, such a fracture and sudden peak drop after the first force are not observed.

Structures with a wall thickness of 0.8 and 1.2 mm in grid-type patterns are also structures with 10% and 20% densities, respectively. In these structures, fracture is more dominant than plastic deformation. For 1.6 mm and 2.0 mm thicknesses with 30% and 40% infill ratios, respectively, plastic deformation is much more dominant. The structure with a 0.8 mm wall thickness and 10% percent infill ratio fractured completely after the first peak force, and the force value instantaneously approached zero in the force–displacement curve.

In the patterns with a gyroid structure, fracture deformations are observed in the model with a 10% infill ratio, while the amount of plastic deformation increases as the infill ratio increases. It can be said that the dominant parameter of these patterns is the infill ratio. Especially in the structure with a 40% infill ratio, fracture started at the end of crushing, and plastic deformation is observed until that moment.

In honeycomb-type patterns, even the structure with the lowest infill ratio shows very little fracture. Especially, the structure with a 40% infill ratio is completely subjected to plastic deformation without any fracture. This can also be seen in the force–displacement graphs of the structures with 30% and 40% infill rates, where the force value continues to increase without decreasing after the first peak force value.

The significant fluctuations observed in the force–displacement curves of thick-walled specimens (e.g., GRID_T20F40D20 and HCOMB_T20F20D24) can be attributed to the intrinsic characteristics of the fused deposition modelling (FDM) process. With a wall thickness of 2.0 mm, corresponding to approximately five perimeter layers, the structures act as a stack of bonded shells. Under compressive loading, stress accumulates until a critical level is reached, at which point multiple interlayer bonds fail simultaneously. This sudden and collective loss of structural continuity leads to the sharp force drops recorded in the curves.

### 3.2. Taguchi Method

In the previous section, comments are made on deformation modes and impact force–crushing displacement curves. In this study, since four different parameters, namely infill pattern, infill ratio, wall thickness, and inner diameter, are examined and the designs are made according to the Taguchi method, the effects of these parameters can be revealed more clearly with the Taguchi method. In this section, the effects of these four parameters on PCF, EA, and SEA will be analysed.

The Taguchi method, developed by Genichi Taguchi, is a robust design optimisation technique widely used to improve product and process performance with minimal experimental effort. This statistical approach, based on the use of orthogonal arrays, systematically reduces the number of experimental trials required while retaining the ability to evaluate the effects of multiple factors and their interactions. The main advantage of the Taguchi method lies in its ability to identify effective design parameters and determine their optimal levels using Signal-to-Noise (S/N) ratios, which measure the sensitivity of performance metrics to external disturbances and variation sources.

To assess the robustness of each parameter combination with respect to the performance indicators considered in this study, namely Peak Crushing Force (PCF), energy absorption (EA), and specific energy absorption (SEA), S/N ratios are computed based on Taguchi’s loss functions. There are two basic optimisation approaches most used in the Taguchi method: “smaller-the-better”, which is appropriate for criteria that are more favourable when minimised, and “larger-the-better”, which applies to criteria that improve as they increase.

In this study, the following evaluations are conducted:

- PCF is evaluated using the “smaller-the-better” approach to reduce the peak force transmitted during impact.
- EA and SEA are evaluated using the “larger-the-better” approach to maximise energy dissipation and mass efficiency.

The S/N calculation equations for “smaller-the-better” and “larger-the-better” are given in Equations (1) and (2), respectively:

(1) $$S/N=-10\log_{10}\left(\frac{1}{n}\sum_{i=1}^{n}y_{i}^{2}\right).$$

(2) $$S/N=-10\log_{10}\left(\frac{1}{n}\sum_{i=1}^{n}\frac{1}{y_{i}^{2}}\right).$$

where *y_i_* is the measurement value of each repetition, and *n* is the number of repetitions.

**Figure 8:**
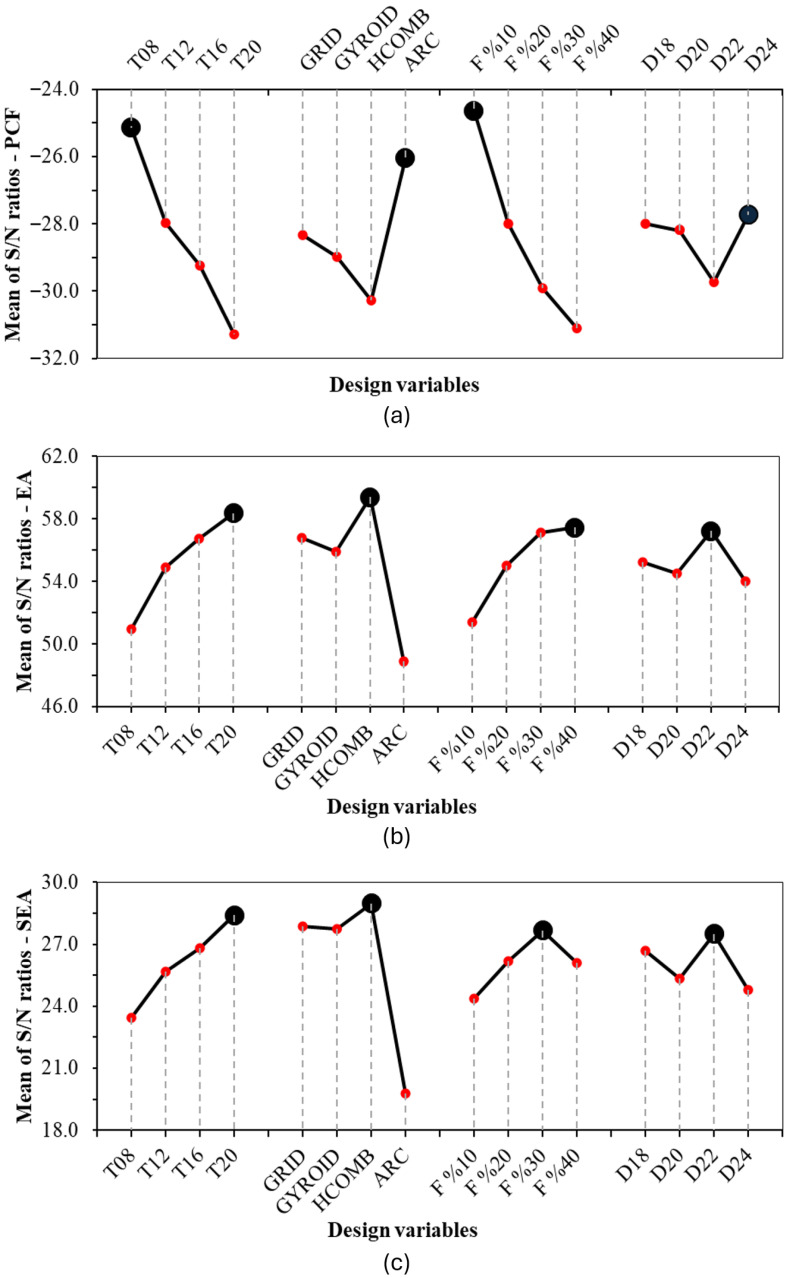
The main effect plots. (**a**) PCF. (**b**) EA. (**c**) SEA.

The crashworthiness criterion values and S/N ratios of bamboo-inspired circular tubes and main effect plots for PCF, EA, and SEA are given in Table 4 and Figure 8, respectively. In the Taguchi method, the optimal level for each parameter in the table of the main effects is the value at the top of the graph. In the main effect graph, the parameter with the largest range for each criterion is the most important parameter. The following explanations will be given with this in mind.

When the effects of the parameters on PCF are analysed, it is seen that the least effective parameter is the infill type, after inner diameter. While the most effective parameter is the infill rate, the wall thickness is also effective to a close level. For PCF to be low, the cross-sectional area must be small. This is also supported by Taguchi’s results. For the smallest PCF, the smallest wall thickness of 0.8 mm and the lowest infill ratio of 10% give the best value. In the infill type, it is the “Archimedean chords”-type infill that is subject to immediate fracture, while there is no definite value for the inner diameter.

An examination of the EA values reveals that the inner diameter, as in PCF, has the smallest effect. The most influential parameters are the fill ratio and wall thickness. While the infill type appears effective, “Archimedean chords” exhibited the worst effect because they fractured immediately. Other infill types are similarly effective. For the best EA value, the cross-sectional area is expected to be as large as possible, resulting in plastic deformation rather than fracture and increasing energy absorption. The wall thickness of 2.0 mm and the infill ratio of 40% support this explanation for the best EA. In the infill type, honeycomb is the best, while grid and gyroid perform similarly.

The parameters have the same effect as EA except for the 40% effect rate in SEA, which is the ratio of absorbed energy to weight. The best infill rate is 40% in EA and 30% in SEA. This shows that the increase in energy absorption value does not compensate for the increase in weight with an increase in the overfill ratio.

When the parameter-specific results are analysed, it is seen that the least effective parameter in PCF, EA, and SEA is the inner diameter. In the infill type, the pattern with the worst performance is “Archimedean chords”. Grid and gyroid have a very close effect, while honeycomb is better in EA and SEA, and PCF is slightly behind the others. Both wall thickness and infill ratio have a very similar effect on each parameter. Only the fill ratio of 40% is worse in SEA than 30%.

### 3.3. ANOVA of Crashworthiness Criteria

In this study, an analysis of variance (ANOVA) is performed using Minitab online to evaluate the statistical significance of four different manufacturing parameters (wall thickness, infill pattern, infill density, inner diameter) on three different performance metrics (Peak Crushing Force—PCF; energy absorption—EA; specific energy absorption—SEA). Each factor is fixed at four levels, and the analyses are conducted across a total of 16 experiments (with two replications). All ANOVA results are summarised in Table 5, Table 6 and Table 7, with detailed interpretations for each response variable provided below.

The analysis of variance performed on PCF revealed that all four factors investigated had a statistically significant effect (*p* < 0.05). Infill density emerged as the most dominant factor on PCF, with the highest F-value (F = 67.28). This is followed by wall thickness (F = 47.91) and infill pattern (F = 29.41). The effect of the inner diameter is more modest compared to the others, yet it remained statistically significant (F = 4.24; *p* = 0.019). Furthermore, the relatively low pure error variance (1.46) indicates that the experimental conditions are well-controlled and that repeatability is at an acceptable level.

The analysis for the EA metric revealed that the relative order of factor importance differed from that of PCF. Infill pattern had the strongest effect on EA (F = 56.01), followed by infill density (F = 40.18) and wall thickness (F = 25.78). The effect of the inner diameter is again statistically significant but considerably weaker than the others (F = 3.75; *p* = 0.029). A statistically significant lack-of-fit is also present in this model (*p* < 0.001). However, the fact that the mean square of pure error (567) remains quite low in absolute value is a strong indicator of good repeatability in the experimental process.

The findings for SEA clearly show that the infill pattern also plays an extremely dominant role for this performance metric (F = 88.33). While wall thickness (F = 26.93) and infill density (F = 26.10) exhibited similar and quite strong effects, the influence of the inner diameter became more pronounced and reached a higher level of significance for SEA compared to the other two metrics (F = 8.15; *p* = 0.001). As with all models, a significant lack-of-fit is present here as well. In contrast, the extremely low pure error variance (0.65) confirms that this experimental study is conducted with exceptionally good repeatability and precision.

In summary, the ANOVA results demonstrated that while all the parameters studied are statistically significant, the dominant factor differed for each performance metric. Infill density is the foremost factor for PCF, whereas infill pattern is identified as the most critical parameter for both EA and SEA. Additionally, the consistently low pure error values across the entire experimental set prove the high repeatability and reliability of the experimental methodology.

**Figure 9:**
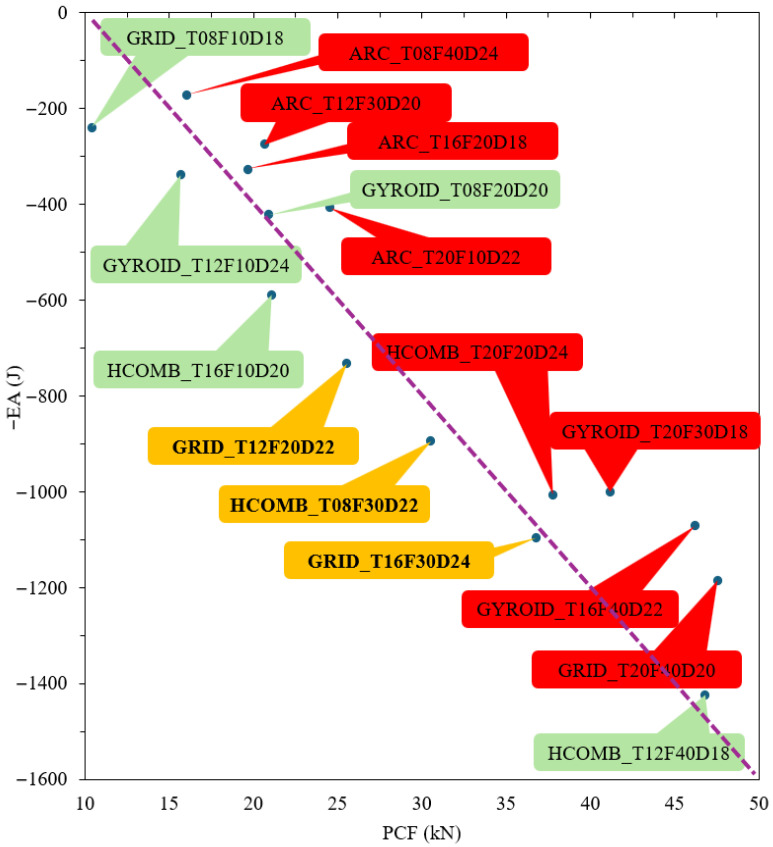
Visualisation of Taguchi experimental design according to PCF and EA criteria.

Figure 9 presents a two-dimensional scatter plot of the PCF and EA crashworthiness criteria, visualising the results of the Taguchi experimental design. A modified Pareto front approach is employed, where EA values are normalised (multiplied by −1) to position the ideal scenario—simultaneously low PCF and high EA—in the lower-left corner, defined as the utopia point.

While Archimedean chords infill structures consistently achieve low Peak Crush Force, their tendency to fracture rather than crush leads to low energy absorption, positioning them as the least efficient designs clustered in the undesirable upper-left region of the graph.

In stark contrast, the three designs closest to the utopia point are highlighted in orange, identifying them as the top performers with an optimal balance of high EA and manageable PCF. These are GRID_T16F30D24, HCOMB_T08F30D22, and GRID_T12F20D22. A second performance tier is marked in green, while lower-performing designs are shown in red.

This visualisation confirms that the grid and honeycomb (Hcomb) infill types are the most ideal structures, effectively dominating the Pareto-optimal frontier. An analysis of these top performers indicates that superior crashworthiness is achievable by combining these robust infill types with an optimal wall thickness (ranging from 8 mm to 16 mm in this study) and an infill ratio of either 20% or 30%. These specific parameter combinations demonstrate the highest potential for creating structures that excel in both key crashworthiness metrics, successfully balancing the trade-off between absorbing energy and controlling impact forces.

### 3.4. Interaction of Material Behaviour and Bio-Inspired Design

The selection of PLA as the base material inherently influenced the observed failure modes, particularly in configurations with low wall thickness and low infill density, where brittle fracture dominated. This raises an important question regarding whether the bamboo-inspired geometry can compensate for the inherent limitations of PLA.

The results indicate that the bio-inspired design—specifically robust infill patterns like honeycomb and grid at optimal infill ratios (20–30%)—effectively mitigates some brittleness by promoting stable, progressive collapse through controlled buckling and localised plastic deformation. These geometric principles help distribute stress and minimise sudden failure initiation, highlighting the potential of bamboo-inspired architectures to enhance energy absorption even in brittle polymers.

Nevertheless, the ultimate performance is constrained by the intrinsic toughness of PLA. It is hypothesised that applying these optimised geometries to tougher, more ductile polymers such as PETG, ABS, or composite filaments would further improve crashworthiness metrics, including specific energy absorption and structural stability. Future work will experimentally validate this by producing the top-performing geometries in tougher polymers to quantify the synergistic effect of bio-inspired design and material selection.

## 4. Conclusions

In this study, a systematic and multi-parametric investigation into the crashworthiness of bamboo-inspired 3D-printed tubes is presented, explicitly extending beyond the conventional focus on infill pattern and infill ratio. Wall thickness and inner diameter were integrated as key design variables—inspired by the graded and porous microstructure of bamboo—and it is demonstrated that these geometric parameters exert a significant and often comparable influence on energy absorption performance. Within the scope of this investigation, the specimens were determined by the Taguchi method, and the structures were manufactured using a 3D printer. The produced models were subjected to compression experiments under quasi-static conditions, and their crashworthiness performance was evaluated. An analysis of variance (ANOVA) was performed to statistically quantify the significance of the design parameters. In line with the obtained data, the effects of the design parameters were analysed according to PCF, EA, and SEA criteria using the Taguchi method. The main outputs of this study are given below:

- Among the parameters of infill type, infill ratio, wall thickness, and inner diameter, the two most effective parameters on crashworthiness performance are infill ratio and wall thickness.
- The Archimedean chords-type infill pattern is the pattern with the lowest performance in terms of crashworthiness performance.
- The grid and gyroid infill patterns perform very similarly to each other, while the honeycomb pattern is better in EA and SEA and slightly worse in PCF.
- EA performance improves as the infill rate increases, but the gain in EA decreases as the infill rate approaches 40%. This is clearly demonstrated by the fact that the SEA value at 30% is superior to that at 40%.
- The Archimedean chords fill type, which exhibits an inconsistent collapse mechanism due to weak connections between the inner walls, showed significant differences between repeated test data and demonstrated low repeatability performance.

While the bamboo-inspired design successfully mitigates some brittle failure modes of PLA by promoting stable deformation, the inherent toughness of the base polymer remains a limiting factor for ultimate performance. The identified geometric principles are expected to be transferable and potentially more beneficial when applied to tougher, more ductile materials, representing a promising direction for future research. It is also important to note that the effects of the parameters reported in this study are specific to the selected parameter ranges. Different outcomes may be observed if different ranges are chosen. Consequently, future studies can focus on producing higher-performance impact-absorbing structures by further optimising wall thickness and infill ratio for a selected optimal infill type, as well as investigating the crashworthiness characteristics of these designs under different compression angles.

## Figures and Tables

**Table 1:** Design parameters and levels used in this experimental study.

Model Parameters	Level 1	Level 2	Level 3	Level 4
Wall thickness (mm)	0.80	1.20	1.60	2.00
Infill pattern	Grid	Gyroid	Honeycomb	Archimedean chords
Infill density (%)	10	20	30	40
Inner diameter (mm)	18	20	22	24

**Table 2:** Taguchi L16 orthogonal experimental design.

Model No	Model Name	Wall Thickness (mm)	Infill Pattern (-)	Infill Density (%)	Inner Diameter (mm)
1	GRID_T08F10D18	0.80	grid	10.00	18.00
2	GYROID_T08F20D20	0.80	gyroid	20.00	20.00
3	HCOMB_T08F30D22	0.80	honeycomb	30.00	22.00
4	ARC_T08F40D24	0.80	Archimedean chords	40.00	24.00
5	GRID_T12F20D22	1.20	grid	20.00	22.00
6	GYROID_T12F10D24	1.20	gyroid	10.00	24.00
7	HCOMB_T12F40D18	1.20	honeycomb	40.00	18.00
8	ARC_T12F30D20	1.20	Archimedean chords	30.00	20.00
9	GRID_T16F30D24	1.60	grid	30.00	24.00
10	GYROID_T16F40D22	1.60	gyroid	40.00	22.00
11	HCOMB_T16F10D20	1.60	honeycomb	10.00	20.00
12	ARC_T16F20D18	1.60	Archimedean chords	20.00	18.00
13	GRID_T20F40D20	2.00	grid	40.00	20.00
14	GYROID_T20F30D18	2.00	gyroid	30.00	18.00
15	HCOMB_T20F20D24	2.00	honeycomb	20.00	24.00
16	ARC_T20F10D22	2.00	Archimedean chords	10.00	22.00

**Table 3:** Printing conditions for PLA tubes.

Operating Parameters	Unit	PLA
Nozzle temperature	°C	220
Baseplate temperature	°C	55
Outer wall print speed	mm/s	200
Inner wall print speed	mm/s	300
Initial layer speed	mm/s	50
Initial layer infill speed	mm/s	105
Extrusion width	mm	0.4
Nozzle diameter	mm	0.4
Initial layer height	mm	0.2
Line width	mm	0.4
Outer wall line width	mm	0.4
Inner wall line width	mm	0.4

**Table 4:** Crashworthiness criteria values and S/N ratios of bamboo-inspired circular tubes.

Model Name	PCF (kN)			EA (J)			SEA (J/g)		
	Exp. 1	Exp. 2	S/N	Exp. 1	Exp. 2	S/N	Exp. 1	Exp. 2	S/N
GRID_T08F10D18	10.55	10.24	−20.34	237.36	240.89	47.57	14.25	14.45	23.14
GYROID_T08F20D20	20.93	20.85	−26.40	417.60	424.90	52.49	19.03	19.24	25.64
HCOMB_T08F30D22	30.23	30.82	−29.69	897.02	890.65	59.02	29.25	29.01	29.29
ARC_T08F40D24	15.01	17.08	−24.12	163.85	178.29	44.64	5.83	6.34	15.66
GRID_T12F20D22	25.66	25.42	−28.14	727.64	735.72	57.29	27.27	27.62	28.77
GYROID_T12F10D24	15.56	15.81	−23.91	331.70	344.95	50.58	16.16	16.73	24.32
HCOMB_T12F40D18	45.98	47.59	−33.40	1422.78	1423.57	63.07	33.21	33.18	30.42
ARC_T12F30D20	23.46	17.85	−26.38	294.53	253.78	48.69	9.96	8.58	19.27
GRID_T16F30D24	36.88	36.70	−31.31	1081.23	1111.65	60.80	31.50	32.34	30.08
GYROID_T16F40D22	46.17	46.19	−33.29	1080.91	1059.89	60.59	28.39	27.81	28.97
HCOMB_T16F10D20	21.25	20.91	−26.48	575.64	601.41	55.39	21.97	22.85	27.00
ARC_T16F20D18	18.38	20.98	−25.90	331.35	321.97	50.28	11.59	11.25	21.15
GRID_T20F40D20	47.24	47.87	−33.54	1166.47	1203.03	61.47	29.16	30.03	29.42
GYROID_T20F30D18	41.27	41.09	−32.29	1009.67	990.90	60.00	40.08	40.05	32.06
HCOMB_T20F20D24	38.08	37.44	−31.54	1004.64	1009.35	60.06	28.64	28.76	29.16
ARC_T20F10D22	24.53	24.57	−27.80	462.05	352.05	51.95	16.45	12.52	22.98

**Table 5:** ANOVA results for PCF.

Source	DF	Adj SS	Adj MS	F-Value	*p*-Value
Wall thickness	3	1397.48	465.83	47.91	0
Infill pattern	3	857.81	285.94	29.41	0
Infill density	3	1962.33	654.11	67.28	0
Inner diameter	3	123.55	41.18	4.24	0.019
Error	19	184.72	9.72		
*→ Lack-of-fit*	3	161.38	53.79	36.88	0.000
*→ Pure error*	16	23.34	1.46		
Total	31	4525.89			

**Table 6:** ANOVA results for EA.

Source	DF	Adj SS	Adj MS	F-Value	*p*-Value
Wall thickness	3	936,992	312,331	25.78	0
Infill pattern	3	2,035,309	678,436	56.01	0
Infill density	3	1,460,357	486,786	40.18	0
Inner diameter	3	136,117	45,372	3.75	0.029
Error	19	230,161	12,114		
*→ Lack-of-fit*	3	221,087	73,696	129.95	0.000
*→ Pure error*	16	9074	567		
Total	31	4,798,936			

**Table 7:** ANOVA results for SEA.

Source	DF	Adj SS	Adj MS	F-Value	*p*-Value
Wall thickness	3	501.46	167.15	26.93	0
Infill pattern	3	1645.05	548.35	88.33	0
Infill density	3	486.03	162.01	26.1	0
Inner diameter	3	151.79	50.6	8.15	0.001
Error	19	117.95	6.21		
*→ Lack-of-fit*	3	107.50	35.83	54.85	0.000
*→ Pure error*	16	10.45	0.65		
Total	31	2902.28			

## Data Availability

The original contributions presented in this study are included in the article. Further inquiries can be directed to the corresponding author.
